# Cushing's Syndrome due to Ectopic ACTH from Bronchial Carcinoid: A Case Report and Review

**DOI:** 10.1155/2012/215038

**Published:** 2012-05-17

**Authors:** Manohara Kenchaiah, Steve Hyer

**Affiliations:** Department of Endocrinology, Epsom and St Helier University Hospitals NHS Trust, Surrey SM5 1AA, UK

## Abstract

Despite advances in analytic and imaging techniques, the syndrome of ectopic adrenocorticotrophic hormone (ACTH) secretion from a tumour resulting in Cushing's syndrome continues to pose difficult diagnostic and therapeutic challenges. Dynamic testing may be equivocal and radiology indeterminate. We report a patient presenting with Cushing's syndrome associated with ectopic ACTH secretion from a bronchial carcinoid whose management presented diagnostic and therapeutic challenges.

## 1. Introduction

The association between cancer and Cushing's syndrome was first recognized in 1928 in a patient with small cell carcinoma of the lung [1]. However, it was not until the 1960s that ACTH was shown to be produced by nonpituitary tumours [2]. Although initially the syndrome was largely associated with small cell lung cancer, the spectrum of cancers is now recognised to include carcinoid tumours especially of the lungs, thymus, and gastrointestinal tract, islet cell tumours, phaeochromocytomas, and medullary thyroid carcinomas [3]. In several large series, ectopic ACTH secretion accounts for approximately 10% of Cushing's syndrome [4]. We describe a patient with ectopic ACTH from a bronchial carcinoid tumour highlighting the unusual presentation and difficulties in management.

## 2. Case Report

**Figure 1:**
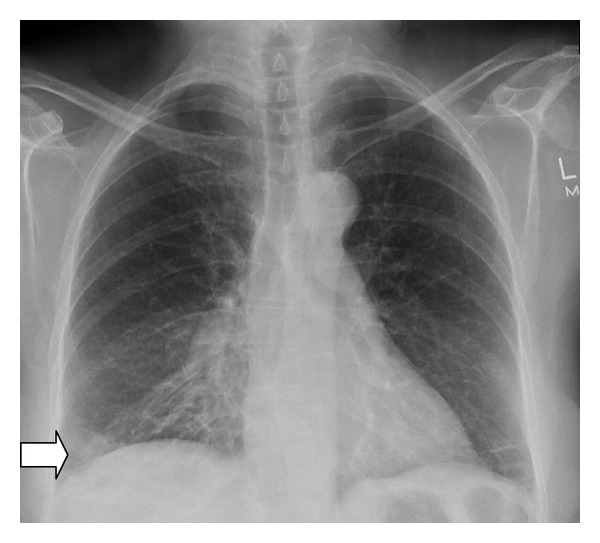
PA chest X-ray showing ill-defined opacity right lower zone (arrow).

A 61-year-old lady with a past medical history of breast cancer presented with facial swelling and a typical Cushingoid appearance which had become apparent for about a year prior to presentation. She was noted to be mildly hypertensive, centrally obese, normoglycaemic, and normokalaemic. Her 9:00am plasma cortisol was 1652 nmol/L (reference: 180–800 nmol/L) and plasma ACTH 454 ng/L (reference < 50 ng/L). Her plasma cortisol failed to suppress after overnight 1 mg dexamethasone (412 nmol/L) or after 8 mg dexamethasone (499 nmol/L) (normal suppression < 50 nmol/L). She declined inferior petrosal sinus testing. An extensive search was undertaken to establish the source of ACTH. Chest X-ray revealed a long standing small peripheral nodule in the right lower lobe which had hardly changed over six years (Figure 1). MRI pituitary scan was normal. CT scans of the chest, abdomen, and pelvis were largely unremarkable apart from some mild adrenal hyperplasia bilaterally. No abnormal octreotide uptake was demonstrated on whole body ^111^In-octreotide scintigraphy. A gut peptide screen was unremarkable. An FDG-PET scan did not show abnormal activity in the lung lesion or elsewhere.

She was commenced on oral metyrapone 500 mg 8th hourly but promptly became extremely breathless over the next few weeks. Urgent chest X-ray showed fluffy opacities throughout both lungs. She was reviewed by a chest physician who found her to be hypoxic. Urgent bronchoscopy and bronchoalveolar lavage revealed increased neutrophils but no abnormal cells or bacteria. Immunological tests were unremarkable. She was felt to have respiratory failure secondary to an acute phase diffuse interstitial alveolitis, probably unmasked by treatment of her Cushing syndrome. She was not deemed fit enough for nodule biopsy due to her breathing difficulties. The alveolitis gradually resolved with supportive measures, and she subsequently underwent bilateral laparoscopic adrenalectomy after discussion at multidisciplinary team (MDT) meeting. She tolerated the procedure well and thereafter commenced hydrocortisone and fludrocortisone replacement.

**Figure 2:**
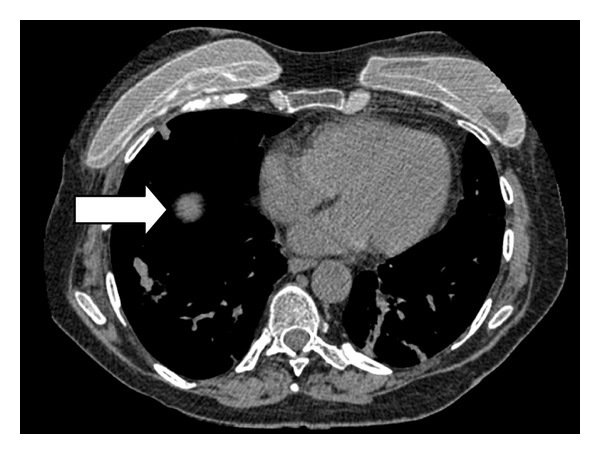
CT chest showing right lower lobe pulmonary nodule (arrow).

**Figure 3:**
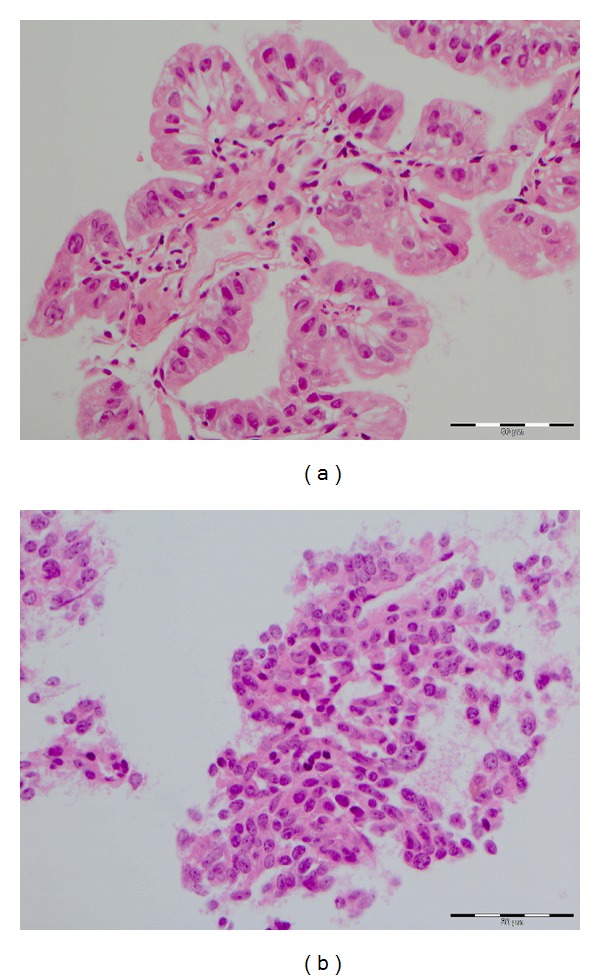
Histology of pulmonary specimen. H&E staining. Original magnification ×200. Histology demonstrates typical cuboidal cells with granules. Specific immunostaining (not shown) was strongly positive for ACTH, CD56, chromogranin and synaptophysin.

A CT-guided biopsy of her right lung nodule (Figure 2) was eventually performed when her respiratory function had recovered sufficiently and staining strongly for ACTH, CD56, chromogranin and synaptophysin (Figures 3(a) and 3(b)). There were no features of metastatic breast cancer.

Following thoracotomy and resection of her lung lesion, the plasma ACTH decreased significantly (preop: 454 pmol/L; postop 25 pmol/L), and she remains well on maintenance steroids. When reviewed in clinic 12 months after surgery, she was symptomatically well, with good lung function.

## 3. Discussion

The differential diagnosis of Cushing syndrome and in particular differentiation of pituitary Cushing syndrome (Cushing disease) from an ectopic ACTH secreting neoplasm can be difficult [5]. Typically patients with ectopic ACTH production have high ACTH levels (>20 ng/L), cortisol levels fail to be suppressed with high doses of dexamethasone (8 mg/day) and demonstrate absent pituitary adrenal responses to corticotropin-releasing hormone (CRH) [6]. However, this is not always unequivocal as 20–40% of patients with ectopic ACTH demonstrates cortisol suppression on high-dose dexamethasone and 10–15% responds to CRH stimulation [6]. The most useful test is inferior petrosal sinus sampling where patients with pituitary lesions show a gradient in ACTH concentration between the affected side sinus and the periphery in contrast to an ectopic ACTH syndrome when there is no gradient [7]. Unfortunately our patient initially declined this investigation and subsequently was too unwell to undergo this examination.

Of the common causes of ectopic ACTH, small cell lung carcinoma makes up about 27%, bronchial carcinoids 21%, islet cell tumours of the pancreas (16%), and thymic carcinoids 10% [3]. Bronchial carcinoids typically have a long history and slow onset of symptoms (1–84 months, median 23.6) so that by the time they present with clinical symptoms attributable to lung disease, classical Cushingoid features are usually present [3]. Our patient had symptoms of cortisol excess for at least 12 months prior to presentation. In most cases, patients with bronchial carcinoids do not have respiratory symptoms or signs making them hard to detect clinically.

Localisation of the source of ectopic ACTH can be problematic. The lung is the most likely organ to harbour an ectopic source of ACTH, being the origin of over 45% of tumours followed by thymus (11%) and pancreas (8%) [3, 5]. Small peripheral bronchial carcinoids can easily be missed on CT chest scans due to poor inspiratory effort, abdominal fat, and basal atelectasis; tumours as small as 0.5 mm may produce florid Cushing's syndrome [8]. Meticulous attention to technique during CT is essential. Using a modern multidetector high-resolution CT able to acquire images 16–24 slices per second, 2.5 mm slices from lung apex to iliac crests is recommended [9]. MRI and octreotide scintigraphy are of little value to identify small bronchial carcinoids [9]. Serial CT and MRI scans fail to localize around 33%–44% of ectopic corticotropin-producing tumours [8, 10, 11]. In our patient a peripheral lung nodule had been noted for some six years but had not changed in appearance over this time and hence initially was not thought to be clinically relevant.

Biochemical clues may also be useful in directing attention to the relevant site for scanning [12]. Elevated calcitonin suggests medullary thyroid cancer, whilst abnormal gut peptides (pancreatic peptide, somatostatin, VIP) suggest a gastrointestinal or pancreatic source. Similarly abnormal catecholamines suggest an adrenal medullary source. Pancreatic islet cell tumours and medullary thyroid cancer associated with ectopic ACTH secretion are usually large and have already metastasized to the liver by the time Cushing's syndrome is diagnosed [3]. In such tumours Cushing's is not usually the main complaint on presentation [3].

Confirmation of ectopic ACTH production requires demonstration of immunostaining positive for ACTH in the resected tumour. This may be difficult if the source was metastatic malignancy as in this case only a subpopulation of the cells will produce ACTH making it difficult to be demonstrated by staining. More recently, extraction of appropriate mRNA by real time PCR provides a highly specific means of identifying these tumours [3].

Management of patients with ectopic ACTH requires control of the hypercortisolaemia as soon as the diagnosis is established [9]. Ketoconazole and metyrapone have good evidence for their efficacy and safety [13, 14]. Patients with identifiable sources of ectopic ACTH should have the tumours resected. Surgery may be curative in more than 80% of  bronchial carcinoids [9]. However, even with modern techniques, a source of ectopic ACTH may still not be found in as many as 12% of patients [9]. Such cases with “occult” ACTH-secreting tumours remain challenging and may require repeat investigating over many years. In these cases, bilateral adrenalectomy, preferably performed laparoscopically, represents a therapeutic option. It is effective treatment with low morbidity and mortality in experienced hands [15]. However, these patients will require long-term glucocorticoid and mineralocorticoid replacement. In addition, they must be counselled regarding the risks of adrenal insufficiency.

We are unaware of a similar case where a diffuse interstitial alveolitis followed treatment of Cushing syndrome. A hypersensitivity reaction to metyrapone might have involved the lungs but this seems unlikely as the condition improved despite continuation of the drug. It seems more likely that the condition was suppressed by the patient's hypercortisolaemia and flared up as the cortisol levels were normalised.

Advances in medical and surgical treatments have improved the overall survival rate for patients with ectopic ACTH. Prognosis depends on the primary tumour histology. Patients with small cell lung carcinoma (SCLC) had the worst prognosis, usually dying within 12 months of diagnosis (median 6–8 months) [9, 15, 16]. Patients with bronchial carcinoids have the best prognosis and are usually considered to have low to moderate grade malignancy. They do have a malignant potential, however, and can be associated with metastases.

## 4. Conclusion

Ectopic ACTH-secreting tumours present some of the most challenging differential diagnoses in endocrinology and require careful clinical, biochemical, radiological, and pathological investigation. These tumours are best managed in a multidisciplinary setting with close liaison between the endocrinologist, endocrine surgeon, chemical pathologist, and radiologist.
